# Assessing the impact of prescribed medicines on health outcomes

**DOI:** 10.1186/1743-8462-4-1

**Published:** 2007-02-15

**Authors:** Wayne D Hall, Jayne Lucke

**Affiliations:** 1 School of Population Health, University of Queensland, Herston QLD, 4006, Australia; 2 Population Health and Uses of Medicines Unit, University of New South Wales, Sydney, Australia

## Abstract

This paper reviews methods that can be used to assess the impact of medicine use on population health outcomes. In the absence of a gold standard, we argue that a convergence of evidence from different types of studies using multiple methods of independent imperfection provides the best bases for attributing improvements in health outcomes to the use of medicines. The major requirements are: good evidence that a safe and effective medicine is being appropriately prescribed; covariation between medicine use and improved health outcomes; and being able to discount alternative explanations of the covariation (via covariate adjustment, propensity analyses and sensitivity analyses), so that medicine use is the most plausible explanation of the improved health outcomes. The strongest possible evidence would be provided by the coherence of the following types of evidence: (1) individual linked data showing that patients are prescribed the medicine, there are reasonable levels of patient compliance, and there is a relationship between medicine use and health improvements that is not explained by other factors; (2) ecological evidence of improvements in these health outcomes in the population in which the medicine is used. Confidence in these inferences would be increased by: the replication of these results in comparable countries and consistent trends in population vital statistics in countries that have introduced the medicine; and epidemiological modelling indicating that changes observed in population health outcomes are plausible given the epidemiology of the condition being treated.

## Background

Many developed countries publicly subsidize selected medicines on the assumption that their use will improve the health of patients who take these drugs [1]. Improved health outcomes might include: reduced incidence of disease (if medicines prevent new cases of disease in persons at risk); reduced mortality and morbidity (if medicines are used to treat early or established cases of a disease); and reduced morbidity and disability or improved quality of life (if medicines are used to slow the progression or palliate the symptoms of an established disease).

Drugs are usually only subsidised if they have been shown to be safe and effective in randomised controlled trials (RCTs). Although data from RCTs provides good reasons for expecting that widely prescribed medicines may improve health outcomes, the improved health outcomes observed in such trials may not occur in routine clinical use. Controlled clinical trials may provide optimistic estimates of effectiveness under routine clinical care because the medicines are used to treat more seriously ill patients in the community than were studied in clinical trials [2,3]. Drugs may also not be prescribed, they may be prescribed inappropriately, and patients may not comply with recommended use of the drugs.

This paper evaluates the research methods that can be used to assess whether the use of prescribed medicines improves health outcomes in the community. It reviews methods used to make causal inferences about the relationships between medicine use and health outcomes (e.g. [4,5]).

## Making causal inferences

When we say that medicine use is a cause of an improved health outcome we mean that it is a contributory cause of the improved health outcome in the sense that use of the medicine is one of a complex set of conditions that jointly produced the improvement in health. In order to infer that medicine use is a contributory cause of an improved health outcome we need evidence that: (1) medicine use and the health outcome covary; and (2) evidence that makes other explanations of the relationship implausible, leaving medicine use as a plausible contributory explanation of the improved health outcome [6-8].

### Assessing covariation

We can assess whether medicine use and a health outcome covary in experiments (such as randomised controlled trials) or in observational studies (e.g. ecological, case-control, cohort, time series and cross-sectional studies).

### Excluding alternative explanations

A and B may be correlated without being causally related. Hence, in order to make a case for a causal relationship we need to exclude plausible alternative explanations of the relationship [7-9]. Experiments provide the strongest basis for excluding alternative explanations of covariation but they are expensive and difficult to conduct. Observational designs are easier to enact but provide a weaker warrant for causal inferences because of their limitations in excluding the following alternative explanations [8,10].

#### Chance?

We can assess the plausibility of chance by constructing a confidence interval around the measure of covariation between medicine use and the health outcome. If the confidence interval does not include the value consistent with the absence of a relationship between medicine use and the health outcome, then we can infer that medicine use and a health outcome covary [11,12].

#### Cause or consequence?

If medicine use is a cause of an improved health outcome, then medicine use should occur before the improvement. Experiments and cohort studies (which measure medicine use before assessing the health outcome) provide the best basis for deciding which is cause and which is consequence [3].

#### A common cause?

If medicine use and the health outcome covary, and medicine use precedes the improved health outcome, we then have to exclude the possibility that a common cause explains the relationship between the two. Experiments provide the best evidence against a common cause because randomisation to an active medicine or a placebo ensures that subjects differ only in whether or not they have been exposed to the medicine [12,13]. When subjects are randomly assigned to a medicine or a placebo then all other causal factors will be equally distributed between the two groups [14] and hence, any difference between the two groups can be attributed to medicine use.

Randomisation is not infallible because there are "threats to validity" that may arise after random assignment that may undermine the equivalence of the two groups. There may, for example, be differential rates of drop-out from the two treatments, or subjects who have been assigned to the control treatment may obtain active treatment elsewhere [12,15]. If there are no such threats to validity, then experiments provide a stronger warrant for causal inferences than observational studies because the former exclude more alternative explanations than the latter.

## Causal inferences from observational data

When experiments and intervention studies cannot be done for ethical and practical reasons common causes must be excluded by indirect means. The logic of the approach is conceptually straightforward: we see whether A and B covary when possible common causes are statistically "controlled for".

One approach to this goal is to control potentially confounding variables in the ***study design***. For example, we could rule out the hypothesis that any relationship between non-steroidal anti-inflammatory drug (NSAID) use and coronary heart disease was a consequence of concurrent medicine use by: (i) excluding individuals with the disease who used other medicines from a cohort study; (ii) by matching cases and controls on concurrent medicine use; or (iii) by stratifying potential participants in a cohort study on confounding variables and matching on those variables [3,16].

Another commonly used approach to dealing with confounding in epidemiology is ***covariate adjustment***. In this approach, all study participants are measured on potentially confounding variables (covariates) and statistical methods are used to estimate the association between A and B while controlling for the covariates [16-19].

***Propensity score analysis ***can be used to assess the plausibility of selection bias as an explanation of relationships in observational studies where patients select their own treatment [13,16]. In this approach, covariates are used to predict the exposure condition that each individual had the greatest propensity to receive. The resulting "propensity score" can be used either as a matching variable or as a covariate in regression analyses [13,20].

***Sensitivity analyses ***can be used when we do not have measures of potential confounders for covariate adjustment or propensity score analysis. Such analyses explore the plausibility of confounding as an explanation of observed outcomes [13,16,21]. These analyses involve modelling the relationship between medicine use and the health outcome under various scenarios in which a confounding variable is related in varying degrees to both medicine use and the outcome. If the relationship between the two persists when allowance is made for plausible degrees of confounding then we can be more confident that the relationship is likely to be causal ([22], pp 193–196).

The major limitation of all these strategies is that they can only rule out specified alternative hypotheses. That is, we have to identify a candidate common cause that we can then match on, measure and adjust for using covariate adjustment or propensity scores, or model in sensitivity analyses. Randomisation is superior to all these strategies because it rules out all possible common causes, including ones that have not been measured or thought of [12].

## Making causal inferences about medicine use and health outcomes

In making causal inferences about relationships between health outcomes and the use of medicines we need evidence that they covary, and if they do, we need to exclude alternative explanations of the covariation, other than that medicine use is a contributory cause of the improved health outcome. In assessing covariation we are asking whether there has there been a change for the better in a health outcome that is related to medicine use. If health outcomes have improved as medicine use has increased then we need to assess (a) whether there is a statistically reliable relationship between the two and if so, we need to (b) quantify the magnitude of the relationship.

If there is an association between medicine use and improved health outcomes, we then have to evaluate alternative explanations of any relationship that we have observed. If we fail to find a relationship between a population health outcome and medicine use, we need to evaluate alternative explanations of why a relationship may not have been observed before accepting the hypothesis that medicine use failed to improve population health outcomes.

Our capacity to make these inferences depends upon the type of data that are available to assess covariation and the plausibility of alternative explanations. A major distinction can be made between covariation observed in two types of data: linked data on medicine use and health outcomes in individuals; and aggregate data on medicine use in a population and population health outcomes.

## Casual inferences from individual data on health outcomes and medicine use

Ideally we would examine relationships between medicine use and health outcomes in large samples of individuals who comprised a representative sample of the population about which we wish to make inferences. These data may be collected in very large-scale special-purpose longitudinal studies of representative samples of the population (e.g. [23]), but such studies are very expensive to mount and time-consuming to conduct [24]. Unless they collect comprehensive data on health outcomes, they may only able to examine the health outcomes that they were primarily designed to study and even then they often rely upon self-reports of both health outcomes and medicine use.

An alternative approach that has been used in Canada, Europe and the USA [25-30] is to link electronic data on medicine use and health outcomes in identified individuals that is routinely collected in administrative health care databases [31]. These linked databases typically link data on identified individuals in separate data sets, such as, hospital morbidity collections, mortality data, disease registers, records of outpatient care, and records of prescribed medicines.

These data sets are usually linked without individual consent because of the impracticality of obtaining it [32]. A view often taken by research ethics committees is that individual data can only be obtained for research purposes with the consent of the person on whom the data have been collected. This is impractical with large administrative data sets because of the costs and logistical challenges in contacting individuals and the fact that personally contacting individuals to obtain their consent may be arguably more intrusive than using their data without their consent. If ethics committees insist that consent is required to link individual data, then record linkage studies cannot be done on the effects of medicine use on health outcomes. In that case, studies of the benefits of medicine use will be restricted to statistical analyses of aggregate data on medicine use and health outcomes.

These data sets can be linked without individual consent if a mechanism for de-identification has been included in the process. In Australia, for example, such data are classified as 'de-identified' and consent is no longer required for public interest research. A protocol has been designed for the Western Australian data linkage project to permit health data to be linked in ways that are acceptable to ethics committees and consistent with the relevant legislation [32].

Administrative databases may not include data on patient characteristics that predict treatment outcome [33]. Key missing data may include: individuals' use of alcohol and tobacco; individuals' use of over-the-counter medications; and the presence of comorbid conditions that will affect treatment outcome [33]. The latter may have to be assessed indirectly via proxy indicators, such as hospital treatment for a comorbid condition.

The major statistical challenge in studying the benefits of medicine use via linked data is dealing with "confounding by indication" [4,34-36]. Because patients who have particular diseases are more likely to be prescribed medicines, those who receive the medicines usually have an higher risk of experiencing adverse health outcomes that are attributable to their disease, regardless of their treatment, than patients who do not have the disease [35]. If account is not taken of such confounding, then observational studies may misleadingly suggest that the medicine use produces harm when in fact it may be beneficial [20,36,37]. The analytical approaches outlined above can also be used to address confounding in linked data sets, that is, covariate adjustment and analyses using propensity scores, and sensitivity analyses to assess the impact of sample selection bias on relationships between medicine use and health outcomes [16,20,36].

The capacity of covariate adjustment and propensity analysis to adequately control for confounding by indication depends upon the quality of data available in the database on potential confounders, [38] and the extent of the overlap of distributions on key covariates between individuals exposed to the two or more treatments that are being compared [20,36]. A major limitation of both methods is that they are only as good as the covariates that are available to control for confounding. If key covariates that predict the outcome (e.g. tobacco and alcohol use, comorbid conditions, etc) are not measured then neither of these approaches can be used to control for confounding by indication [36,38]. In the absence of measures of key potential confounders, we are limited to sensitivity analyses and epidemiological modelling to assess the seriousness of the threat that confounding by indication poses to the validity of any inferences that can be draw from the data on the benefits of medicines [16].

## Causal inferences from aggregate data on health outcomes and medicine use

When individual linked data on pharmaceutical use and health outcomes are not available we can only assess associations between (1) population data on pharmaceutical use and (2) population health outcomes such as mortality or morbidity attributable to a specific disease [5]. The analysis of aggregate data on medicine use and health outcome comprises a type of "ecological analysis" that uses data on groups to make inferences about the health of individuals [39,40]. If we assume, in the absence of good reasons for so doing, that individual level relationships can be inferred from aggregate level relationships, then we are said to have committed the "ecological fallacy" [41,42].

The dominant view in the epidemiological literature is that ecological studies should only be conducted when individual level data are unavailable. Even then they are only seen as providing, at best, inexpensive and relatively efficient ways of generating hypotheses that need to be tested in analyses of relationships between these variables measured in individuals (e.g. [5,10,39,43]). According to the approach adopted here, we need to identify the major threats to the validity of inferences from aggregate to individuals and then either design our studies to avoid them or to analyze the data in ways that minimize these errors.

### Assessing changes for the better in a health outcome

Vital statistics on population mortality and treated morbidity that are collected as a standard part of public health surveillance in most developed countries can be used to monitor trends in population health [44,45]. Disease case registers (e.g. of cancer mortality or incidence, cardiovascular disease, diabetes) provide trend data on disease incidence and prevalence for a population using standardised criteria for defining cases. Registry data improve upon vital statistics by producing time series data on disease incidence or prevalence that use consistent diagnostic criteria.

Trends in the prevalence of self-reported morbidity, degree of disability and quality of life can be estimated from periodic cross-sectional surveys of large representative samples of the population. These data provide cross-sectional estimates of the prevalence of self-reported health status. Sometimes they also collect data on self-reported use of medicines [45].

### Measuring medicine use in the population

Trends in medicine use can be inferred from aggregate pharmaceutical data. These may comprise data on the sales by volume or formulation of a specific drug or a drug class. Sales data do not provide any information on who is being prescribed the medicine (unless they are only prescribed to people within a narrow age range or to a single sex). They also do not tell us who is having the drugs dispensed and who is complying with the recommended use of the medicines.

Data on the number of scripts that are written or dispensed are closer to medicine use than sales data but they usually do not include information on patient diagnoses or on patient compliance. Even limited information on the characteristics of those who are prescribed a drug (such as age and sex) improves on aggregate prescription data because it increases our capacity to study covariations between population medicine exposure and population health outcomes.

## Assessing relationships between aggregate medicine use and health outcomes

### Regression approaches

Regression models can be used to model relationships between temporal trends in population health outcomes and population medicine use. The strongest examples of such analyses are those in which the design enables the researcher to demonstrate a dose-response relationship between medicine use within subsets of the population and health outcomes (e.g. [46]), or between different populations with different levels of exposure to the medicines (e.g. [47]).

### Interrupted time series analysis

Aggregate data on mortality and medicine use each comprise a time series. The health outcomes time series may consist, for example, of monthly mortality from a specific cause in 5-year age groups in a population over a number of years while the medicine use time series may consist of monthly sales data or prescription numbers for a drug or a drug class over the same time period. Interrupted Time Series (ITS) analysis is a set of statistical methods that can be used to assess the impact of an intervention (such as the introduction of a new medicine) on a health outcome time series [48-50]. The onset of the intervention is usually specified as the date when a new drug was introduced into a market.

A family of statistical methods can be used to analyze the effects of an intervention on time series data while taking account of autocorrelation: correlations between data at different time points that invalidates conventional statistical methods such as ordinary linear regression [51,52]. ITS analyses may involve using segmented linear regression methods [48,52] or Auto-regressive Integrated Moving Average (ARIMA) models [51]. More recently, econometricians have used generalized least squares or partial likelihood methods to fit more complex models to time series data [49]. All such statistical methods enable the effect of the intervention to be separated from general trends and serial dependencies in time so that valid statistical inferences can be made about whether an intervention has had an effect on the time series [50].

## Alternative explanations of improved population health outcomes

If improved population health outcomes and population medicine use are associated, we need to assess whether the association can be plausibly attributed to other factors. A widely used strategy for causal inference in this situation is quasi-experimentation [12,15]. In quasi-experimentation, the investigator first generates and then evaluates plausible alternative explanations of the relationships that have been observed with the aim of arriving, by a process of elimination, at a conclusion that medicines use in the population is the most plausible explanation of the improved population health outcomes [12,53].

### Changes in measurement

The first alternative hypothesis that needs to be considered is that changes in population health outcomes reflect changes in the way that the health outcome has been measured. This could occur as a result of a change in the system of classification (e.g. the edition of the International Classification of Disease) that is used to code cause of death or morbidity, a change in diagnostic criteria, or increased attention to some causes of deaths that may increase the apparent rate of deaths attributable to that cause and reduce the rates of other causes of death [12,44,48]. These possibilities need to be excluded by evidence that coding systems, diagnostic criteria or medical scrutiny have remained stable over the study period. If a change in measurement remains a plausible hypothesis, its effects may be assessed by sensitivity analyses.

### Confounding factors

Rarely is a change in medicine use the only change that has occurred during the period of interest that may affect the population health outcome we are studying. Concurrent historical events that affect the health outcome are another plausible hypothesis that needs to be excluded [48]. One plausible alternative explanation is that improved population health outcomes may reflect a declining prevalence in the population of major risk factors for a disease. Another possibility is that changes in population health outcomes may be due to the effects of the increased use of other medicines or medical interventions in the population.

## Assessing the plausibility of alternative explanations

We can assess the plausibility of alternative explanations by conducting further analyses of data and by citing supportive data from other studies. If we have measures of population exposure to other medical interventions, or aggregate data on population trends in risk factors, we can assess the plausibility of these explanations by regression analyses (e.g. [54,55]). Sensitivity analyses can also be conducted using the study data to evaluate the plausibility of alternative explanations [16,21].

We can also look for supporting evidence from other studies. This evidence may include the *absence *of similar trends in population health outcomes in populations that have not introduced the medicine. The presence of a similar relationship occurring at different times in countries that have introduced the medicine at different dates, and its absence in countries that have not done so, reduces the plausibility of alternative hypotheses (e.g. [47]). Our confidence in the impact of medicine use on population health outcomes will increase with replications of the relationship between medicine use and health outcomes in different countries which introduce the medicine at different times.

### The role of epidemiological modelling

Epidemiological models of a disease implemented in computer programs can be used to assess the plausibility of alternative explanations of improvements in population health outcomes. Macrolevel epidemiological models model the mortality of hypothetical populations (e.g. 1000 adults aged 35–39 years) from a starting age a year at a time until a specified age (e.g. 75 years). Empirical probabilities of experiencing different health states are derived from population data on disease incidence and mortality. Data from clinical trials or observational studies on the effectiveness of interventions are used to predict the effects of interventions on mortality and morbidity in the cohort. Markov models are used to model transitions between different disease states over the lifetime of the cohort of individuals [56].

Microlevel models attempt to simulate the lifetime mortality experience of large samples of individuals who vary in characteristics that predict the risk of developing a disease, dying from the disease or another cause, and responding to interventions. These models sum the estimated effects on individuals to predict the effects of interventions on population health outcomes [56]. Such models require more knowledge of the natural history of the disease, the effects of different interventions on its natural history, and relationships between individual characteristics and disease risk and treatment outcome. They can also be computationally complex.

Sensitivity analyses are used to assess the robustness of the results of both types of modelling. In macro-level models, sensitivity analyses evaluate the impact of uncertainty about key model parameters on the model's predictions about population health outcomes [56]. In the case of microlevel models, sensitivity analyses assess the effects of different starting assumptions on the results of modelling.

Epidemiological models can be used to assess the likelihood that observed trends in disease incidence or mortality can be attributed to an intervention. A good example of the utility of this type of epidemiological modelling is provided by studies that assessed the plausibility of the hypothesis that a decline in prostate cancer mortality in the USA in the early 1990s was due to the widespread adoption of prostate specific antigen (PSA) screening in that country in the late 1980s (e.g. [57,58]).

## Explaining a lack of improvement in population health outcomes

If there is no improvement in a population health outcome after the introduction of a medicine we need to consider a number of possible explanations for failures to find an association [59].

First, a drug may not have been used by a large enough proportion of the patient population to produce a detectable improvement in the population health outcome. Estimates may be obtained from epidemiological modelling of the likely effects of medicine use on population health outcomes for (i) varying percentages of patient coverage and (ii) varying estimates of the amount by which the efficacy observed in clinical trials is reduced in routine clinical use.

Second, we need to consider whether our study has had sufficient statistical power to detect improvements in health outcomes of the size that we expect (given the probable efficacy of the medicine and its likely patient coverage). Statistical power will depend upon both the size of the expected impact on population health outcomes and the rate of increase in medicine use. It may be difficult to detect changes in population health outcomes if there is a slow incremental increase in medicine use. It may be easiest to detect an effect if there is a steep, substantial and sustained increase in medicine use for a condition for which no effective treatment previously existed.

Third, if a large proportion of eligible patients in the population are receiving the medicine we also need evidence that the drug is being prescribed to patients who will benefit from it and that these patients are taking the drug in the required dosages with the required frequency.

Fourth, we need to consider the possible impact of countervailing factors on population health outcomes. These may include worsening trends in risk factors that offset any population health benefits or the effects of other medications.

## Conclusion

There is no "gold standard" method for assessing the impact of medicine use on health outcomes. In its absence, a convergence of evidence from different types of studies using multiple methods of independent imperfection provides the best reason for attributing improvements in health outcomes to the use of medicines (see table 1).

**Table 1:** Approaches to studying the effects of medicines on health outcomes

Method of study	Strengths	Limitations
Randomised Controlled Trials and meta-analyses of such trials	• Gold standard evidence for causal relationship by virtue of randomisation to treatment	May not predict effects of medicines on health outcomes because: • May be too small to detect rare adverse events • May be too short to detect long term adverse effects • May exclude high risk patients e.g. those with comorbidity • May involve optimal treatment and compliance
Linked data on individuals	• Links data on medicine use and health outcomes in individuals • Closer to routine clinical practice than evidence from RCTs • Cheap and quick to do retrospectively	• Confounding by indication: patients who use medicines are at a higher risk of a disease • Limited assessment of confounders e.g. comorbidity, OTC drugs, alcohol & tobacco • Often uses treated morbidity as a proxy for comorbidity
Ecological studies	• Simple and cheap to do because use existing data on medicines and health outcomes • Directly examine relationships between population medicine use and health outcomes	• Use aggregate rather than individual level data • Crude measures of medicine use e.g. drug sales or scripts • Limited capacity to exclude alternative explanations such as changes in risk factors, and increased use of other treatments
Epidemiological modelling	• Mathematical synthesis of epidemiological data on the disease and clinical trial data on safety and efficacy of medicines	• Simplifications of complex natural history of disease • Uncertainties about long term effects of medicines (addressed by sensitivity analyses) • Underdeveloped in studies of effects of medicines on health outcomes

The major requirement for being able to do so is good evidence that:

1. a safe and effective medicine is being appropriately prescribed in clinical practice;

2. there is covariation between medicine use and improved health outcomes;

3. we can discount alternative explanations of the covariation, leaving medicine use as a plausible explanation of the improved health outcomes.

The strongest possible evidence for an inference that the use of a medicine has improved population health outcomes would be provided by the coherence of the following types of evidence:

1. Individual linked data showing that patients are prescribed the medicine, there are reasonable levels of patient compliance, and there is a relationship between medicine use and health improvements that is not explained by other factors;

2. Evidence of aggregate improvements in these health outcomes in the population in which the medicine is used;

3. The replication of these results in comparable countries;

4. Consistent trends in population vital statistics in countries that have introduced the medicine;

5. Epidemiological modelling that changes observed in population health outcomes are plausible, given the epidemiology of the condition, and the clinical effectiveness of the medicines (after discounting for the decline in efficacy observed in RCTs to that expected in routine clinical practice).

In the absence of individual linked data the next best evidence comes from the coherence of the following types of evidence:

1. Trends in population health outcomes that covary with rates of exposure to the medicine in defined sub-populations (e.g. age, sex or geographic area);

2. Similar trends in comparable countries that have introduced the medicine;

3. The absence of improvements in mortality in comparable countries that have not introduced the medicine;

4. Evidence from epidemiological modelling that the changes observed in population health outcomes are plausible given the epidemiology of the condition and the clinical effectiveness of the medicines (after discounting for decline of efficacy in routine clinical practice).

Evidence from ecological and small observational studies warrants less confidence in causal inference than data from large-scale linked data sets. It may be the best that is available if the community is unprepared to allow record linkage in the absence of individual consent or governments are unprepared to invest in the necessary infrastructure to permit data linkage on medicines use and health outcomes. These limitations of such data provide a strong case for record linkage.

## Abbreviations

ITS – interrupted time series

NSAID – non-steroidal anti-inflammatory drug

RCT – randomised controlled trial
